# Supplementary material for: Preclinical evaluation of manufacturable SARS-CoV-2 spike virus-like particles produced in Chinese Hamster Ovary cells
Source: Commun Med (Lond). 2023 Aug 23;3:116. doi: 10.1038/s43856-023-00340-7 (PMC10447459; doi:10.1038/s43856-023-00340-7)
Supplement: Supplementary file 2 — Supplementary information [file 43856_2023_340_MOESM2_ESM.pdf]

Preclinical evaluation of manufacturable SARS-CoV-2 spike virus-like particles produced in Chinese Hamster Ovary cells

Supplementary Figures

Sergio P. Alpuche-Lazcano[1], Matthew Stuible[1], Bassel Akache[2], Anh Tran[2], John Kelly[3], Sabahudin Hrapovic[4,] Anna Robotham[3], Arsalan Haqqani[3], Alexandra Star[3], Tyler M. Renner[2], Julie Blouin[1], Jean-Sébastien Maltais[1], Brian Cass[1], Kai Cui[5], Jae-Young Cho[5], Xinyu Wang[5], Daria Zoubchenok[1], Renu Dudani[2], Diana Duque[2], Michael J. McCluskie[2], Yves Durocher[1]*

[1] Human Health Therapeutics Research Centre, National Research Council Canada, 6100 Royalmount Avenue, Montreal, QC, H4P 2R2, Canada.

[2] Human Health Therapeutics Research Centre, National Research Council Canada, 1200 Montreal Road, Ottawa, ON, K1A 0R6, Canada.

[3] Human Health Therapeutics Research Centre, National Research Council Canada, 100 Sussex Dr, Ottawa, ON, K1A 0R6, Canada.

[4] Aquatic and Crop Resources Development Research Centre, National Research Council Canada, 6100 Royalmount Avenue, Montreal, QC, H4P 2R2, Canada.

[5] Nanotechnology Research Centre, National Research Council Canada, 11421 Saskatchewan Drive, Edmonton, Alberta T6G 2M9, Canada.

*** Corresponding author:** Human Health Therapeutics Research Centre, National Research Council Canada, 6100 Royalmount Avenue, Montreal, QC, H4P 2R2, Canada. Electronic address: yves.durocher@nrc.gc.ca.

a)

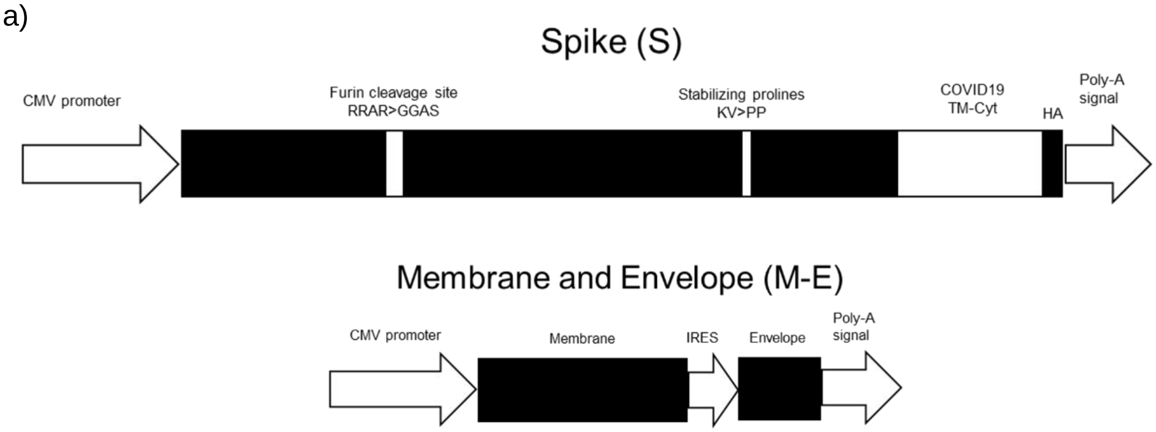

b)

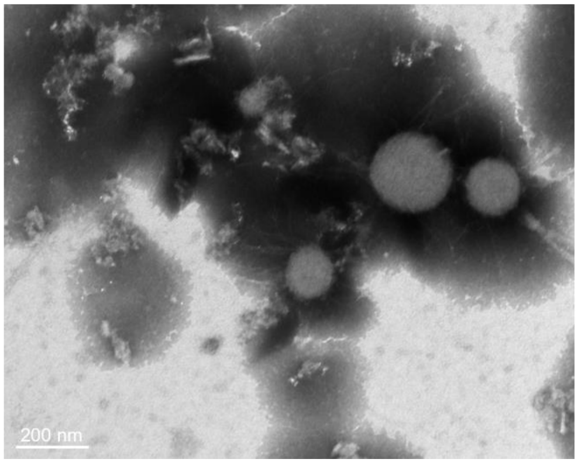

Supplementary Fig. 1: Generation of different S-VLP types. a, Schematics of SARS-CoV-2 M-E and S constructs used in the current study. All S constructs used in this manuscript for ancestral and variants contain the mutations depicted in the “Spike” schematic. **b,** Representative TEM image S-VLPs mock condition. A 200 nm scale bar is shown at the bottom of the image.

a)

b)

c)

d)

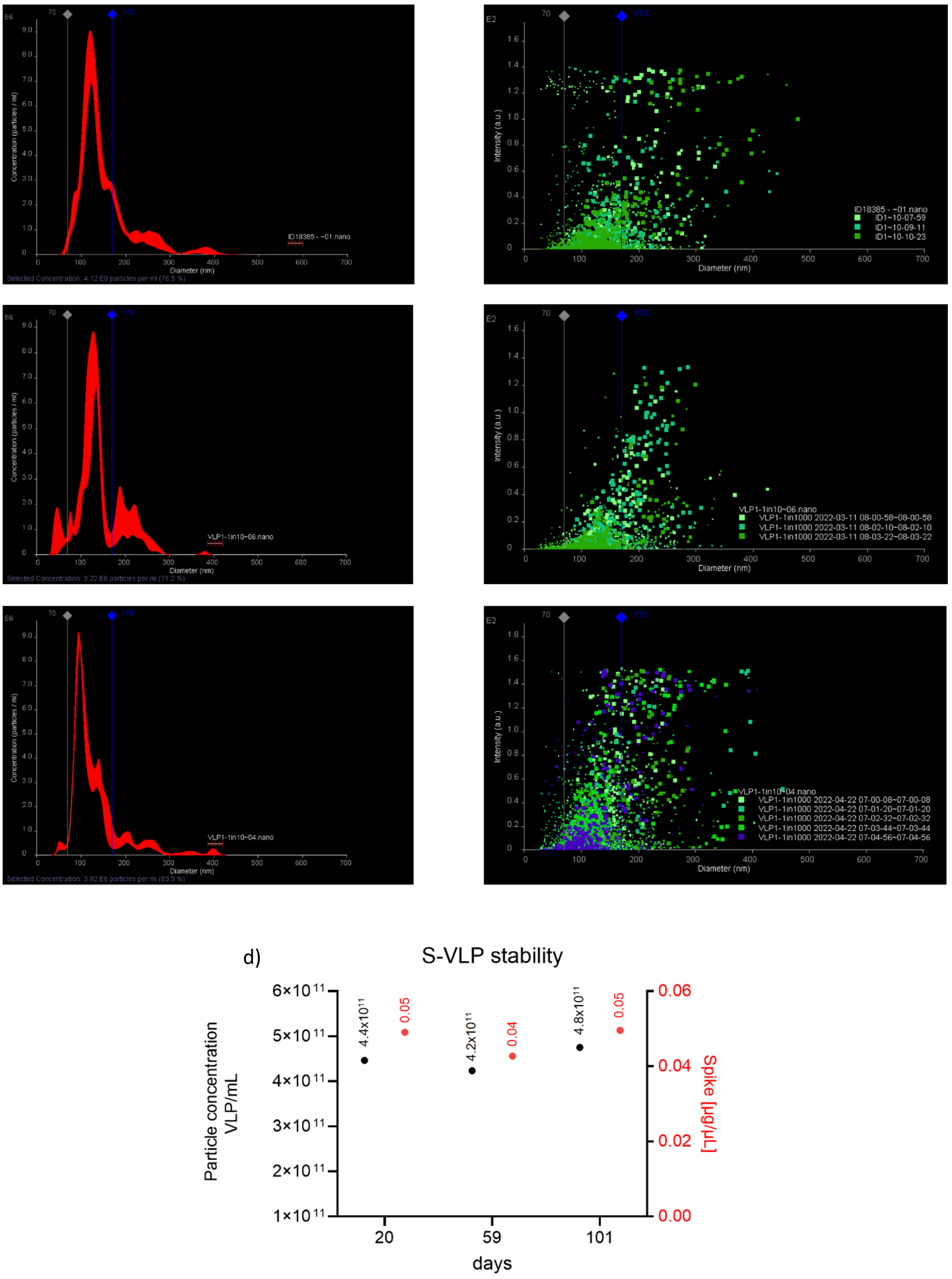

Concentration (particles / ml)
Diameter (nm)
Intensity (a.u.)
ID18385 - ~01.nano
ID1~10-07-59
ID1~10-09-11
ID1~10-10-23
VLP1-1in10~06.nano
VLP1-1in1000 2022-03-11 08-00-58~08-00-58
VLP1-1in1000 2022-03-11 08-02-10~08-02-10
VLP1-1in1000 2022-03-11 08-03-22~08-03-22
VLP1-1in10~04.nano
VLP1-1in1000 2022-04-22 07-00-08~07-00-08
VLP1-1in1000 2022-04-22 07-01-20~07-01-20
VLP1-1in1000 2022-04-22 07-02-32~07-02-32
VLP1-1in1000 2022-04-22 07-03-44~07-03-44
VLP1-1in1000 2022-04-22 07-04-56~07-04-56
S-VLP stability
Particle concentration VLP/mL
Spike [µg/µL]
days
20
59
101
4.4x10¹¹
0.05
4.2x10¹¹
0.04
4.8x10¹¹
0.05

Supplementary Fig. 2: Quantification and size determination of S-VLPs by NTA. a-c, Representative NTA of purified S-VLPs at 1:1000 dilution. VLPs samples were analyzed by NTA at day 20 **(a)**, 59 **(b)** and 101 **(c)** post-production. On the left, finite track length adjustment analyses (FTLA) show concentration vs size. Red shadows represent the ±SEM of 3 different technical measurements per sample. y-axis shows VLP concentration at $x10^6$ particle/mL. 70-170 nm size gates are shown on the top of each plot. On the right, the VLP distribution scatterplot (intensity vs size) displays 70-170 nm size gates. At least three different injections of each sample were analyzed (bottom-right). **d,** S-VLP stability graph. VLP quantities and S concentration were monitored for 101 days in samples stored at 4°C. The left y-axis shows the VLP concentration per mL determined by NTA (black). S concentration (red) is displayed on the right y-axis.

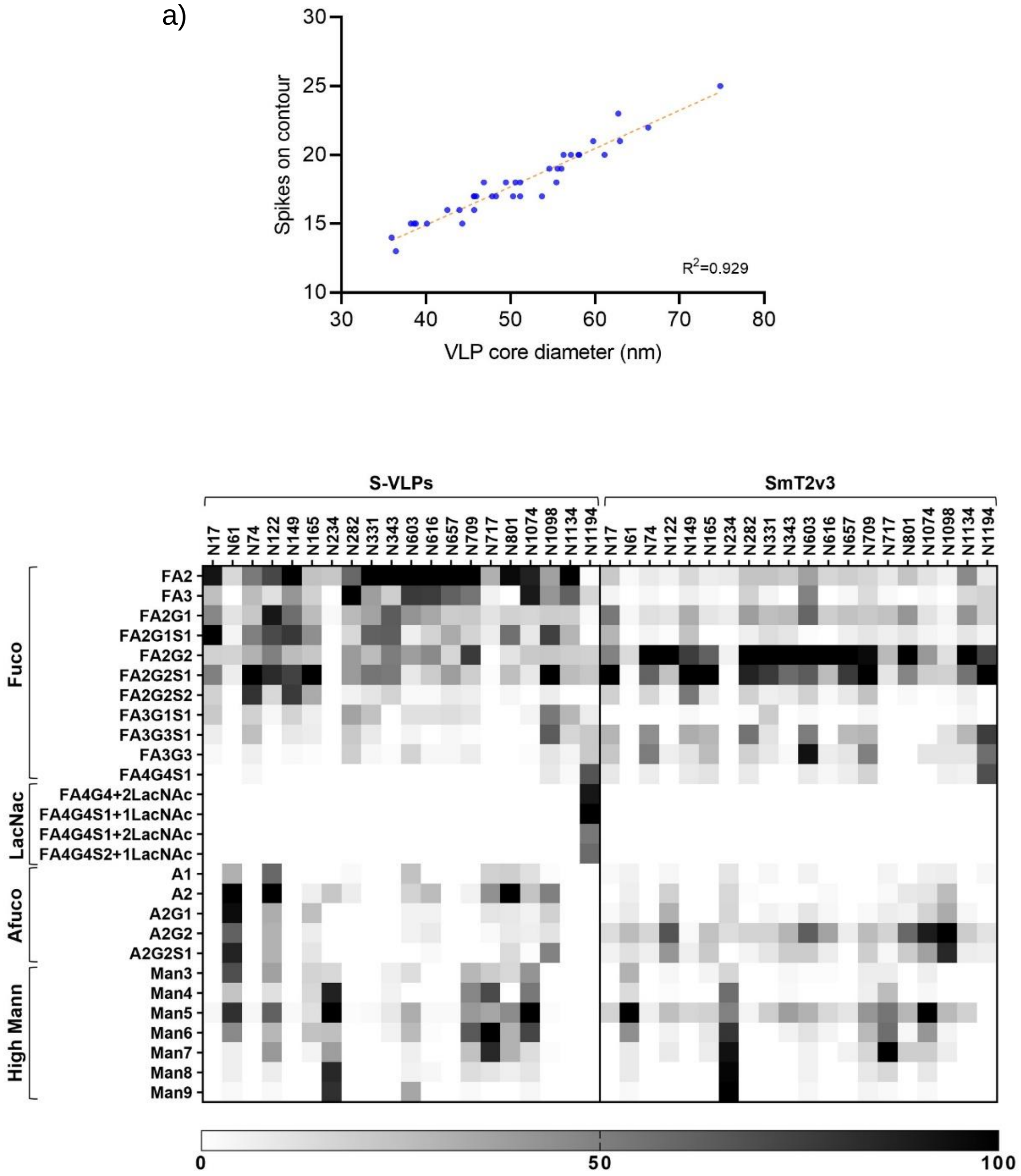

Supplementary Fig. 3: S protein density estimation on particle surface and S glycosylation analysis. **a,** Cryo-EM spike counting on VLP perimeters correlates with core diameters. A linear regression plot of 36 individual VLPs shows a strong correlation between peripherals spikes and core diameters (R= 0.9641 and R^2= 0.9294). **b,** Heatmap illustrating the N-glycosylation of the spike protein**.** VLPs S protein and the soluble form SmT2v3 were subjected to N-glycan identification by LC-MS/MS. Glycosylation was characterized at 20 out of 22 N-glycosylation sites for both proteins. Glycans observed on each site are listed in Oxford notation and are grouped into categories: fucosylated complex-type glycans (Fuco), tetraantennary fucosylated complex-type glycans with evidence of LacNAc extensions (LacNAc), afucosylated complex-type glycans (Afuco) and high mannose glycans (High Mann). Low abundant hybrid glycans are not included. Data for glycans differing in number of sialic acids only are combined. The darkness of the square represents the glycan relative abundance on the site, with the scale bar (0-100) shown below. See also Supplementary Data file.

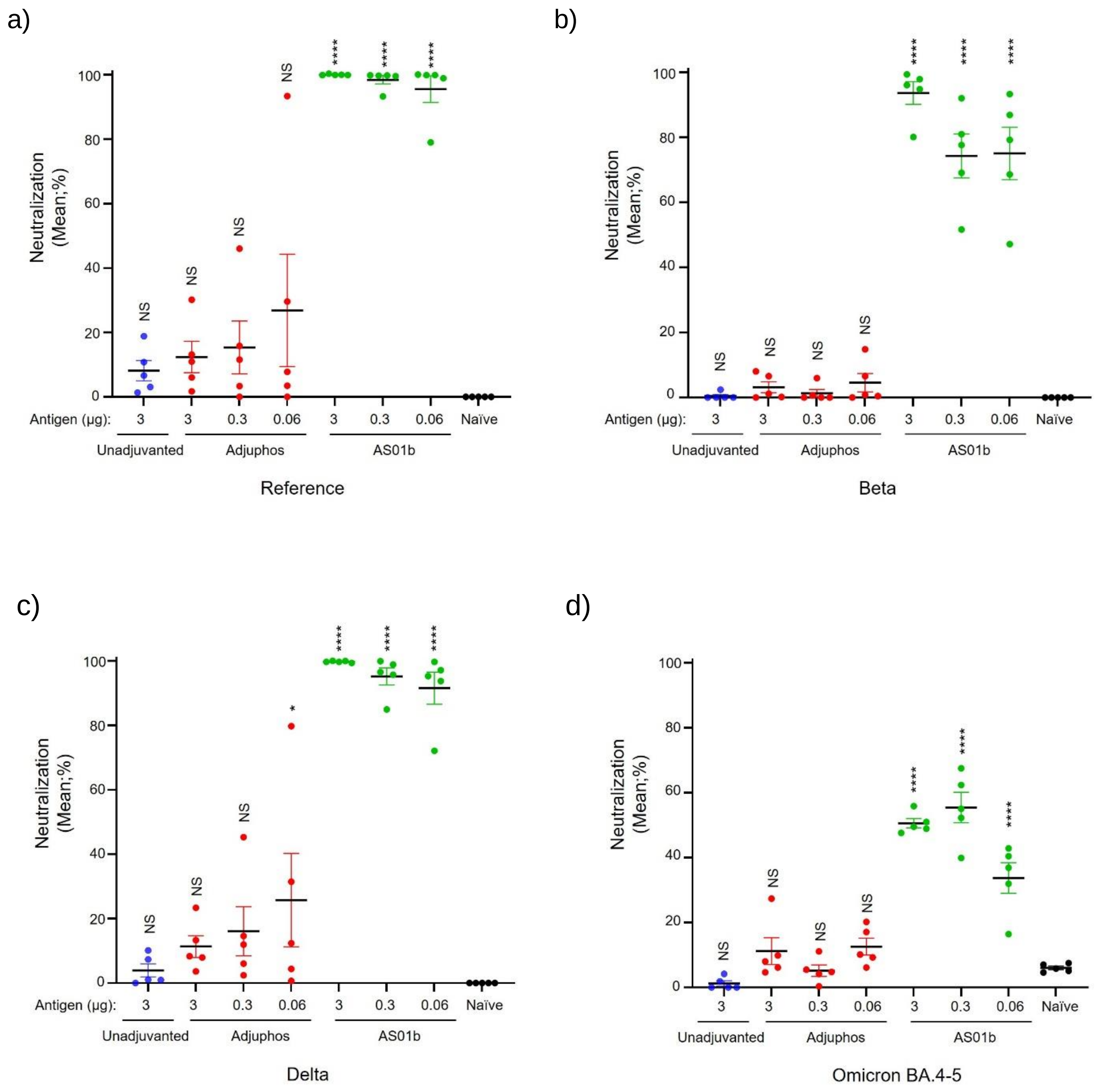

Supplementary Fig. 4: Cell-based (Vero E6) surrogate neutralization assay for SARS-CoV-2 variants. The percentage neutralization mean against SARS-CoV-2 was determined at 1:75 dilution for unadjuvanted and adjuvanted conditions. **a,** Dot plot showing the serum neutralization activity against S from SARS-CoV-2 Reference (ancestral strain, Wuhan) **b,** Beta and **c,** Delta strain **d,** Omicron BA.4-5. Data are presented as mean ±SEM of 5 mice per group. Black dots represent Naïve animals, blue for unadjuvanted, red for Adju-phos and green for AS01b S-VLPs. A one-way ANOVA with Dunnette's multiple comparison test was performed to assess significance. $p \leq 0.0001$ and $p \leq 0.05$ are represented by four and one asterisk respectively and $p > 0.05$ as NS.

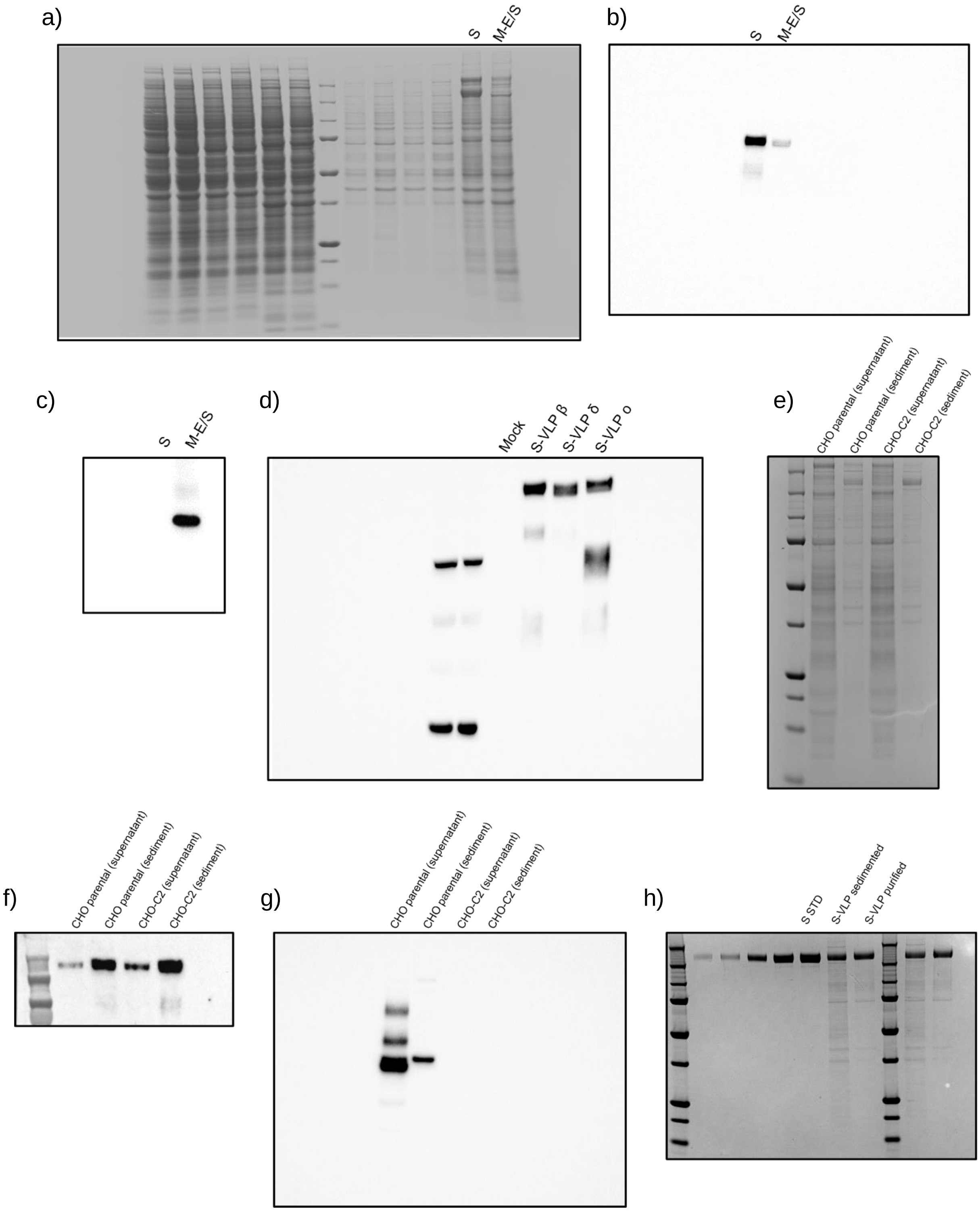

Supplementary Fig. 5: Digital uncropped images of gels and blots. Total protein staining and immunoblots corresponding to **a,** Fig 1a, total protein staining. **b,** Fig 1a, top immunoblot. **c,** Fig 1a, bottom immunoblot. **d,** Fig 1d. **e,** Fig 2a, total protein staining. **f,** Fig 2a, immunoblot, right top. **g,** Fig 2a, immunoblot, right bottom. **h,** Fig 2c.

Supplementary Methods

Sergio P. Alpuche-Lazcano[1], Matthew Stuible[1], Bassel Akache[3], Anh Tran[3], John Kelly[2], Sabahudin Hrapovic[4,] Anna Robotham[2], Arsalan Haqqani[2], Alexandra Star[2], Tyler M. Renner[3], Julie Blouin[1], Jean-Sébastien Maltais[1], Brian Cass[1], Kai Cui[5], Jae-Young Cho[5], Xinyu Wang[5], Daria Zoubchenok[1], Renu Dudani[3], Diana Duque[3], Michael J. McCluskie[3], Yves Durocher[1]*

[1] Human Health Therapeutics Research Centre, National Research Council Canada, 6100 Royalmount Avenue, Montreal, QC, H4P 2R2, Canada.

[2] Human Health Therapeutics, National Research Council Canada, 100 Sussex Dr, Ottawa, ON, K1A 0R6, Canada.

[3] Human Health Therapeutics, National Research Council Canada, 1200 Montreal Road, Ottawa, ON, K1A 0R6, Canada.

[4] Aquatic and Crop Resources Development Research Center, National Research Council Canada, 6100 Royalmount Avenue, Montreal, QC, H4P 2R2, Canada.

[5] Nanotechnology Research Centre, National Research Council Canada, 11421 Saskatchewan Drive, Edmonton, Alberta T6G 2M9, Canada.

*** Corresponding author:** Human Health Therapeutics Research Centre, National Research Council Canada, 6100 Royalmount Avenue, Montreal, QC, H4P 2R2, Canada. Electronic address: yves.durocher@nrc.gc.ca.

Detection of S protein on S-VLP purified samples by ELISA

Purified recombinant reference material hACE2 [1] stock solution (2.05 mg/mL) was diluted in DPBS (without Ca, Mg, and adjusted pH to 7.8) to a final concentration of 2.5 µg/mL. 100 µL of hACE2 dilution was added into 96-well Immulon 4HBX flat bottom plates (Thermo Scientific) followed by an overnight incubation at 4°C. A mock condition (DPBS without hACE2 coating) was also prepared. hACE2 or mock solution was removed and wells were washed with 200 µL of DPBS + Tween-20 (0.05% v/v) (DPBS-T) and blocked with DPBS + BSA (1% w/v) for 1h at RT. Meanwhile, in a 96-well round-bottom 550 µL polypropylene plate (Axygen), S-VLPs ([S]=0.081 mg/mL) were prepared at 20 µg/mL in DPBS + 0.1% Tween + 1% BSA (DPBS-T-B) followed by eleven 2-fold serial dilutions in DPBS-T-B. After a 1h incubation, the blocking solution was removed and 100 µL/well S-VLPs from the dilution plate were added, followed by incubation at RT for 1h. After 1h incubation, the plate was washed with DPBS-T (4x). Next, in-house llama SARS-CoV-2 S antiserum diluted 1:100000 with DPBS-T-B was added (100 µL/well) and incubated for 1h at RT followed by 4X further washes with DPBS-T. After this time, goat anti-llama IgG HRP conjugate (A160-100P, Bethyl) in PBS-T-B (1:4000) was added (100 µL/well) and incubated at RT for 1h. The plate was then washed 4x with DPBS-T and 100 µL/well of tetramethylbenzidine (TMB) was added and incubated in darkness for 30 min. 1 M H2SO4 solution was added to stop the reaction. Optical density at 450 nm was measured using a SpectraMax 340PC (Molecular devices). ELISA results were plotted and analyzed using GraphPad Prism 9.3.1 (GraphPad Software).

S-VLP S quantification and model building

Acquired images by Cryo-EM of rounded S-VLPs (n=36) were individually contrasted using 7x7 light low pass filter and lattice filter to better observe individual S trimers. Then, the spikes on the outer contour were counted and VLP cores delimited by peripheral spikes. Diameter and perimeter of the flat projected area of each core were measured using ImageJ software [2]. To

establish a correlation between spikes and core diameters, a linear regression was plotted using the total number of spikes of the contour and the core diameter on individual particles. With a strong correlation between samples, we developed a VLP model using mySphere(N) function on MATLAB (https://www.mathworks.com). Our model uses the real Euclidian distance between spikes for generating equidistant points depicted with MATLAB-makehgtform function as three-dimensional VLP model. To obtain the real Euclidian distance, we first obtained the arc distance (S) and the projected straight distance between contour spikes (P). Three assumptions were made to relate the arc distance (S) between contour spikes to the real Euclidian distance between any two spikes on the envelope surface: (1) all VLPs are perfect spheres; (2) the spikes are uniformly distributed over the VLP surface; (3) the contour spikes are composed of intercepting and partially overlapping spikes on two different planes. The S distance and the P distance were calculated with the next equation:

$$\mathrm{S} = \frac{\text{Core Perimeter}}{\text{\# of Countour Spikes}} = \frac{\pi \ * \text{Diameter}}{\text{\# of Countour Spikes}}$$

$$\mathrm{P} = \text{VLP Membrane Diameter} * \sin\left(\frac{\mathrm{S}}{\text{VLP Membrane Diameter}}\right)$$

Finally, the real Euclidian distance between spikes (d) was obtained from the following equation:

$$\mathrm{d} = \frac{\mathrm{P}}{\cos\left(\frac{\pi}{4}\right)}$$

Real Euclidian distance values were introduced in MATLAB to generate the locations of spikes on spherical models.

N-Glycosylation of S protein in S-VLPs and soluble S (SmT2v3)

S in-gel digestion

Eight 3.5 µg aliquots of S protein from purified S-VLPs, and as well as eight 3.5 µg aliquots of the recombinant S SmT2v3, were each combined 1:1 with 2 x Laemmli buffer (Bio-Rad) containing 5% β-mercaptoethanol and heated at 95°C for 5 minutes. Aliquots, as well as 10 µL Precision

Plus Protein Unstained Standards (Bio-Rad) were run on two 4-20% Mini-PROTEAN TGX precast 10-well gradient gel (Bio-Rad) using a Mini-PROTEAN 3 electrophoresis cell (Bio-Bad) at 170 V. Gels were stained overnight with Bio-Safe Coomassie Stain (Bio-Rad). Gel bands containing the S protein were excised and destained with 100mM ammonium bicarbonate, 30% acetonitrile. Gel bands were dehydrated with acetonitrile, reduced with 10mM DTT in 50mM ammonium bicarbonate at 56°C for 1h, alkylated with 55mM iodoacetamide in 50mM ammonium bicarbonate at room temperature in the dark for 1h and then treated again with DTT solution to quench any residual iodoacetamide. Half of the gel band replicates for each sample were treated with 2U PNGaseF (Sigma) in 50mM ammonium bicarbonate at 37°C overnight. For each sample, one PNGaseF-treated gel band and one untreated gel band were treated with each of the following proteases in 50mM ammonium bicarbonate: 150 ng trypsin (Promega), 150 ng α-lytic protease (Sigma), 150 ng chymotrypsin (Sigma), or 150 ng Glu-C (Promega). Gel bands treated with chymotrypsin were incubated at 25°C overnight while all other gel bands were incubated at 37°C overnight.

LC-MS/MS

Peptide digests were analyzed on an Orbitrap Eclipse Tribrid mass spectrometer equipped with an electrospray ionization source (Thermo Scientific) connected to an UltiMate 3000 nano-LC system (Thermo Scientific). Injected approximately 1/8 of each protein digest. Reverse phase chromatography was performed on a nanoACQUITY BEH 1.7 µm 100 µm × 100 mm C18 column (Waters) with a C18 PepMap100 5 µm trap (Thermo Scientific). Mobile phase A was 0.1% formic acid in ddH_2O, and mobile phase B was 0.1% formic acid in acetonitrile. Peptides were eluted over a linear gradient of 0.2% to 40% mobile phase B over 89 min, 40% to 95% mobile phase B over 4 min, followed by re-equilibration, with a flow rate of 0.5 µL/min. MS spectra were acquired in the Orbitrap from 350 to 2000 m/z in positive electrospray ionization mode at 120 K resolution. The most intense ions (threshold = 2e6, dynamic exclusion = 25 sec) were selected in the

quadrupole for HCD-MS/MS (isolation window = 1.6 m/z) and a fixed HCD activation energy was selected based on peptide m/z and charge state using a decision tree. MS/MS spectra were acquired in the Orbitrap at 15 K resolution in profile mode. Cycle time was 1 second.

Data analysis

The data files were manually inspected to identify (glyco)peptides covering each putative N-glycosylation site (Supplementary Data file). Peptides covering 2 or more N-glycosylation sites were rejected. Glycopeptide identity was confirmed by manual inspection of the HCD MS/MS spectrum. For each peptide, the retention times of several representative glycopeptides were used to calculate the expected retention time for glycopeptides with a given number of sialic acids. The data was processed using GlycoPIQ v2.1, an in-house software that allows identification and quantification of a large number glycopeptides within nanoLC-MS/MS datasets. Briefly, MS/MS spectra from each nanoLC-MS/MS DDA raw file were centroided and extracted into mzXML file using ProteoWizard msConvert v3.0 [3]. For identification, each MS/MS spectrum was searched using m/z tolerance of 20 ppm against a theoretical spectral library consisting of 2+, 3+ and 4+ ions of each peptide listed in Supplementary Data file modified on the asparagine (N) of the N-glycosylation sequon (NXS or NXT) with each of 398 potential human glycan moieties (listed Supplementary Data file). The library search also included a fixed modification of carbamidomethyl on cysteine residues (+57.02 Da) and variable modifications of deamidation on asparagine residues (+0.984 Da), cyclization to pyro-glutamic acid on N-terminal glutamine residues (-17.027), and cyclization to pyro-glutamic acid on N-terminal glutamic acid residues (-18.011). The glycopeptide identifications and MatchRx software [4] (a module in GlycoPIQ) were used to fine-tune the expected glycopeptide retention times for each sample. For quantification, MatchRx was used to extract the m/z of all hypothetical glycopeptides in the spectral library (m/z tolerance = 10 ppm) in a window +/- 5 min of the glycopeptide expected retention time and calculate the peak area. The sum of the peak areas of 2+, 3+ and 4+ ions was reported as the

raw intensity of the glycopeptide. Glycopeptides for which the raw intensity in the non-PNGaseF-treated samples was less than $2^{0.5}$ times the raw intensity in the PNGaseF-treated sample were removed as likely false positives. The raw intensities were normalized, with the abundance of the glycoform for a given peptide with the greatest intensity set at "100%". The abundance of the unmodified peptide (no glycan) was also presented as relative to the most abundant glycoform and so may have an abundance >100%. The GlycoPIQ results were manually inspected and suspected false positives were confirmed or removed. Final results are shown entirely in Supplementary Data file and include glycoforms that have ≥ 2% relative abundance in at least one of the variants. Glycoforms with a relative abundance <1% are listed with an abundance of 0%.

Mouse serum anti-S ELISA

Briefly, 96–well high-binding ELISA plates (Thermo Fisher Scientific) were incubated overnight at room temperature (RT) with 100 µL of 0.3 µg/mL SMT1-1[5] S protein diluted in PBS. Plates were washed five times with PBS-T 0.05% and blocked for 1h at 37°C with 200 µL of PBS- fetal bovine serum (FBS) 10% (Thermo Fisher Scientific). The plates were washed five times with PBS-T 0.05%. Next, 3.162-fold serially diluted samples in PBS-T 0.05% with 10% FBS were added in 100 µL volumes and incubated for 1h at 37°C. After five washes with PBS-T 0.05%, 100 µL of goat anti-mouse IgG-HRP (1:4,000, Southern Biotech) was added for 1h at 37°C. After five further washes with PBS-T 0.05%, 100 µL/well of the substrate o-phenylenediamine dihydrochloride (OPD, Sigma-Aldrich) diluted in 0.05 M citrate buffer (pH 5.0) was added. Plates were developed for 30 minutes at RT in the dark. The reaction was stopped with 50 µL/well of 4N H_2SO_4. Bound IgG Abs were detected spectrophotometrically at 450 nm. Titers for IgG in serum were defined as the dilution that resulted in an absorbance value (OD 450) of 0.2 and were calculated using XLfit software (ID Business Solutions). Samples that did not reach the target OD were assigned the value of the lowest tested dilution (i.e.10) for analysis purposes.

Cell-based surrogate SARS-CoV-2 neutralization assay

The degree of binding of soluble S protein to the surface of HEK-293T overexpressing human ACE2 or Vero E6 cells is measured following co-incubation of the protein with sera. HEK-293T were maintained in D-MEM high glucose with 10% FBS and 1% penicillin/streptomycin (all from Thermo Fisher Scientific). Vero E6 cells were maintained in RPMI 1640 supplemented with 10% FBS, 1% penicillin/streptomycin, 20mM HEPES, 1x non-essential amino acids, 1x Glutamax, 50µM 2-mercaptoethanol (all from Thermo Fisher Scientific) at 37°C with 5% CO2. Soluble S protein (SmT1) was biotinylated using EZ-Link NHS-LC-LC-Biotin (Thermo Fisher Scientific) according to manufacturer's instructions. Indicated dilutions of mouse serum were mixed with 250 ng of biotinylated S and $1x10^5$ cells in the presence of 0.05% azide in a 96-well V-bottom plate (Nunc, Thermo Fisher Scientific) and incubated for 1h at 4°C, in darkness. Regardless of serum concentration, the final volume of all samples was normalized to 150 µL. Cells were washed with PBS-1% BSA+0.05% azide and incubated with Streptavidin-phycoerythrin conjugate for 1h at 4°C (Thermo Fisher Scientific). After an extra wash, cells were fixed using CytoFix™ (Becton Dickinson) and resuspended in wash buffer + 5mM EDTA for acquisition on an LSR Fortessa (Becton Dickinson). S bound to cells was determined by calculating the geometric Mean Fluorescence Intensity (gMFI) of PE (on singlet cell population) and subtracting the same parameter measured from control cells (incubated in absence of plasma/serum), both above background noise as determined by the negative control, using FlowJo analysis software. Percent neutralization was calculated with the next equation according to [6]:

$$\%\text{neutralization} = \frac{100-[100\text{x}(\text{gMFI of PEsample}-\text{gMFI of PEnegative control})]}{\text{gMFI of PEpositive control}-\text{gMFI of PEnegative control}}$$

Samples with values ≤ 0 were considered 0.

ELISpot

Spleens were mechanically minced and splenocytes were isolated in RPMI containing 10% FBS, 1% penicillin/streptomycin, 1% glutamine (Thermo Fisher Scientific) and 55 µM 2-Mercaptoethanol (Thermo Fisher Scientific). Cells were strained using 70-µm filters and cell concentrations were determined on a Cellometer (Nexcelom). A S peptide library (JPT Peptide Technologies GmbH) consisting of 315 peptides (15mers overlapping by 11 amino acids with last peptide consisting of a 17mer) was used to stimulate the cells. The library was split into 3 sub-pools, each covering a third of the S protein, which were used to separately stimulate 4×10^5 cells in duplicate at a final concentration of 2 µg/mL per peptide. Cells were also incubated without any stimulants to measure background responses. After an incubation of ~20 hours at 37°C with 5% CO_2, plates were washed and developed according to the manufacturer's protocol. AEC substrate (Becton Dickenson) was used to visualize the spots. Spots were counted using an automated ELISpot plate reader (Cellular Technology LTD). For each animal, values obtained with media alone were subtracted from those obtained with each of the S peptide pools, and then combined to yield an overall number of antigen-specific IFN-γ+ SFC/10^6 splenocytes per animal.

References

1. Stocks, B., et al. *ACE2-1: Angiotensin-converting Enzyme 2 Protein Reference Material.* 2022 2022/02/01; Available from: https://nrc-digital-repository.canada.ca/eng/view/object/?id=df43c24e-3213-4f26-94cd-dc273d988f6a.
2. Schneider, C.A., W.S. Rasband, and K.W. Eliceiri, *NIH Image to ImageJ: 25 years of image analysis.* Nat Methods, 2012. **9**(7): p. 671-5.
3. Adusumilli, R. and P. Mallick, *Data Conversion with ProteoWizard msConvert.* Methods Mol Biol, 2017. **1550**: p. 339-368.
4. Haqqani, A.S., J.F. Kelly, and D.B. Stanimirovic, *Quantitative protein profiling by mass spectrometry using label-free proteomics.* Methods Mol Biol, 2008. **439**: p. 241-56.
5. Stocks, B.B., et al., *Characterization of a SARS-CoV-2 spike protein reference material.* Anal Bioanal Chem, 2022: p. 1-9.
6. Akache, B., et al., *Immunogenic and efficacious SARS-CoV-2 vaccine based on resistin-trimerized spike antigen SmT1 and SLA archaeosome adjuvant.* Sci Rep, 2021. **11**(1): p. 21849.
