# Supplementary material for: Preclinical evaluation of manufacturable SARS-CoV-2 spike virus-like particles produced in Chinese Hamster Ovary cells
Source: Commun Med (Lond). 2023 Aug 23;3:116. doi: 10.1038/s43856-023-00340-7 (PMC10447459; doi:10.1038/s43856-023-00340-7)
Supplement: Supplementary file 3 — Description of Additional Supplementary Files [file 43856_2023_340_MOESM3_ESM.pdf]

Description of Additional Supplementary Files

File Name: Supplementary Data 1

Description: Glycosylation dataset

File Name: Supplementary Data 2

Description: Main figures numerical data
